# Advancements and applications of artificial intelligence in cardiovascular imaging: a comprehensive review

**DOI:** 10.1093/ehjimp/qyae136

**Published:** 2024-12-14

**Authors:** Federico Fortuni, Giuseppe Ciliberti, Benedetta De Chiara, Edoardo Conte, Luca Franchin, Francesca Musella, Enrica Vitale, Francesco Piroli, Stefano Cangemi, Stefano Cornara, Michele Magnesa, Antonella Spinelli, Giovanna Geraci, Federico Nardi, Domenico Gabrielli, Furio Colivicchi, Massimo Grimaldi, Fabrizio Oliva

**Affiliations:** Cardiology and Cardiovascular Pathophysiology, S. Maria Della Misericordia Hospital, University of Perugia, Piazzale Giorgio Menghini, 3, 06129 Perugia, Italy; Cardiology Department, Marche University Hospital, Ancona, Italy; Cardiology IV, ‘A. De Gasperis’ Department, ASST GOM Niguarda Ca’ Granda, University of Milano-Bicocca, Milan, Italy; Clinical Cardiology and Cardiovascular Imaging Unit, Galeazzi-Sant'Ambrogio Hospital IRCCS, Milan, Italy; Department of Cardiology, Ospedale Santa Maria Della Misericordia, Azienda Sanitaria Universitaria Friuli Centrale, Udine, Italy; Dipartimento di Cardiologia, Ospedale Santa Maria Delle Grazie, Napoli, Italy; U.O.C. Cardiologia, Azienda Ospedaliero-Universitaria Senese, Siena, Italy; S.O.C. Cardiologia Ospedaliera, Presidio Ospedaliero Arcispedale Santa Maria Nuova, Azienda USL di Reggio Emilia—IRCCS, Reggio Emilia, Italy; U.O.S. Emodinamica, U.O.C. Cardiologia. Ospedale San Antonio Abate, Erice, Italy; S.C. Cardiologia Levante, P.O. Levante—Ospedale San Paolo, Savona, Italy; U.O.C. Cardiologia-UTIC, Ospedale ‘Monsignor R. Dimiccoli’, Barletta, Italy; U.O.C. Cardiologia Clinica e Riabilitativa, Presidio Ospedaliero San Filippo Neri—ASL Roma 1, Roma, Italy; U.O.C. Cardiologia, Ospedale San Antonio Abate, Erice, Italy; S.C. Cardiology, Santo Spirito Hospital, Casale Monferrato, AL 15033, Italy; Department of Cardio-Thoraco-Vascular Sciences, Division of Cardiology, A.O. San Camillo-Forlanini, Rome, Italy; U.O.C. Cardiologia Clinica e Riabilitativa, Presidio Ospedaliero San Filippo Neri—ASL Roma 1, Roma, Italy; U.O.C. Cardiologia, Ospedale Generale Regionale ‘F. Miulli’, Acquaviva Delle Fonti, Italy; Cardiologia 1-Emodinamica, Dipartimento Cardiotoracovascolare ‘A. De Gasperis’, ASST Grande Ospedale Metropolitano Niguarda, Milano, Italy; Presidente ANMCO (Associazione Nazionale Medici Cardiologi Ospedalieri), Firenze, Italy; Consigliere Delegato per la Ricerca Fondazione per il Tuo cuore (Heart Care Foundation), Firenze, Italy

**Keywords:** artificial intelligence, cardiovascular imaging, echocardiography, cardiac computed tomography, cardiovascular magnetic resonance

## Abstract

Artificial intelligence (AI) is transforming cardiovascular imaging by offering advancements across multiple modalities, including echocardiography, cardiac computed tomography (CCT), cardiovascular magnetic resonance (CMR), interventional cardiology, nuclear medicine, and electrophysiology. This review explores the clinical applications of AI within each of these areas, highlighting its ability to improve patient selection, reduce image acquisition time, enhance image optimization, facilitate the integration of data from different imaging modality and clinical sources, improve diagnosis and risk stratification. Moreover, we illustrate both the advantages and the limitations of AI across these modalities, acknowledging that while AI can significantly aid in diagnosis, risk stratification, and workflow efficiency, it cannot replace the expertise of cardiologists. Instead, AI serves as a powerful tool to streamline routine tasks, allowing clinicians to focus on complex cases where human judgement remains essential. By accelerating image interpretation and improving diagnostic accuracy, AI holds great potential to improve patient care and clinical decision-making in cardiovascular imaging.

## Graphical Abstract

**Graphical Abstract:**
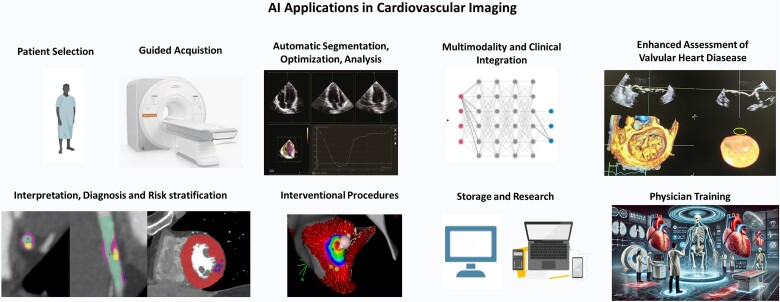

## Introduction

Artificial intelligence (AI) is revolutionizing the field of cardiovascular medicine, offering transformative advancements in the way clinicians acquire, interpret, and integrate imaging data. By reducing image acquisition time and guiding physicians or technicians through the imaging process, AI has streamlined cardiovascular imaging. This guidance can help ensure optimal image quality, even in cases where the operator is less experienced, ultimately reducing variability.^1^ AI’s ability to optimize the vast amounts of data generated from imaging studies also enhances the accuracy and detail of reconstructed images, making it easier for clinicians to extract critical diagnostic information.^2,3^

One of the key innovations AI brings is the ability to facilitate integration between different imaging modality as well as between imaging data and clinical data.^3^ For instance, AI algorithms can cross-reference imaging results from echocardiography, cardiac computed tomography (CCT), or cardiovascular magnetic resonance (CMR) with a patient’s clinical records, lab results, and genetic information.^3^ This integration not only provides a more comprehensive view of the patient condition but also helps in streamlining workflows by organizing vast amounts of information into a more interpretable format.^3^ Moreover, AI aids in the interpretation of images by suggesting potential diagnoses based on patterns identified from large datasets, which may assist physicians in diagnosing complex or rare conditions.^4^ This ability to analyse large datasets can also help in planning patient management strategies by identifying high-risk patients early on, allowing for timely interventions.^5^

From an administrative point of view, AI holds immense potential in organizing and integrating patient data. It can automate the process of gathering and synthesizing patient history, imaging results, and treatment plans, making this information readily available during consultations or follow-up visits. This seamless access to organized data improve clinical efficiency, enabling physicians to make informed decisions quickly and reducing administrative burden.^3^ Moreover, AI’s role in streamlining patient data can improve continuity of care, ensuring that crucial information is always at hand, regardless of when or where the patient is seen.

In research, AI’s ability to analyse imaging data could help physicians to discover unconventional patterns or information that might be overlooked during traditional image interpretation. For instance, subtle markers of disease progression that are not typically recognized may be highlighted through AI’s analytical algorithms. This could aid human interpretation to open new pathways for research and improve early detection of conditions.

In addition to its diagnostic and organizational capabilities, AI is a powerful tool for training physicians. From the image acquisition process to the interpretation of complex data, AI can provide real-time feedback and suggestions, helping less experienced clinicians develop their skills.^1,6^ It can simulate various clinical scenarios, making it an invaluable tool for medical education and ongoing professional development.

In this review, we will explore the clinical applications of AI in cardiovascular imaging, with dedicated sections analysing its use in echocardiography, CMR, CCT, interventional cardiology, nuclear medicine, and electrophysiology (EP). We will highlight its potential benefits and limitations, recognizing that while AI can enhance imaging interpretation and data integration, it cannot replace the expertise of physicians. Rather, AI serves as a tool to make their work faster, more accurate, and more efficient, ultimately enhancing patient care and clinical outcomes.

## Echocardiography

AI has numerous applications in echocardiography, including guided image acquisition for optimal imaging, automated quantification of cardiac function, and disease detection and classification. It can also enhance strain analysis and 3D echocardiography, improve risk stratification, and optimize clinical workflow, potentially leading to faster, more accurate assessments, and streamlined decision-making (*Table 1*).

**Table 1:** Main applications of AI in echocardiography

Application	Description	Benefits
Guided image acquisition	AI guides less experienced operators to acquire images correctly.	Reduces dependency on highly skilled operators, making echocardiography more accessible.
Automated quantification	AI algorithms automatically measure heart chamber sizes and volumes.	Saves time, reduces manual measurement errors, and standardizes measurements.
Disease detection and classification	AI identifies and classifies cardiac diseases such as cardiomyopathies, valve disorders, etc.	Early and accurate disease detection supports decision-making.
Strain analysis	AI conducts strain analysis to assess myocardial function and deformation.	Provides detailed assessment of myocardial health, enhances early detection of dysfunction.
3D echocardiography	AI assists in reconstructing 3D models of the heart.	Provides detailed structural visualization, improves surgical planning.
Risk stratification	AI models predict patient outcomes based on echocardiographic data.	Enhances risk assessment, supports clinical decision-making, and personalizes patient care.
Workflow optimization	Optimize scheduling, reporting, and data management.	Improves efficiency, reduces wait times, and enhances overall patient care quality.

In recent years, research has increasingly focused on the integration of AI into echocardiography to enhance accuracy, reliability, and reproducibility of echocardiographic analysis. The historical main limit of traditional echocardiography is the inter-observer high variability in image acquisition and interpretation. One of the major areas where AI has shown remarkable potential is in the standardization and automation of echocardiographic image acquisition. The quality of the images can significantly be affected by operator ability, and obtaining high-quality echocardiographic images usually requires high experience. Through the development of deep learning-based algorithms, we have now systems capable of guiding the acquisition of high-quality images by automatically adjusting the angle of the ultrasound probe. A prospective study by Narang *et al*.,^1^ for example, recruiting nurses with no experience in echocardiography, demonstrated that even less experienced operators can achieve a level of image quality comparable to that of experienced sonographers, after a trained period with an AI-guided system. Accordingly, in 2020, the Food and Drug Administration approved in the United States of America the first AI-based echocardiographic device to guide clinicians in capturing high-quality images.^7^ This development appears beneficial in particular settings, such as emergency departments, where experienced echocardiographers may not be readily available. Moreover, the manual process of acquisition being time-consuming and subject to human errors should be rapidly solved thanks to this automatic system of analysis and optimization.

**Figure 1:**
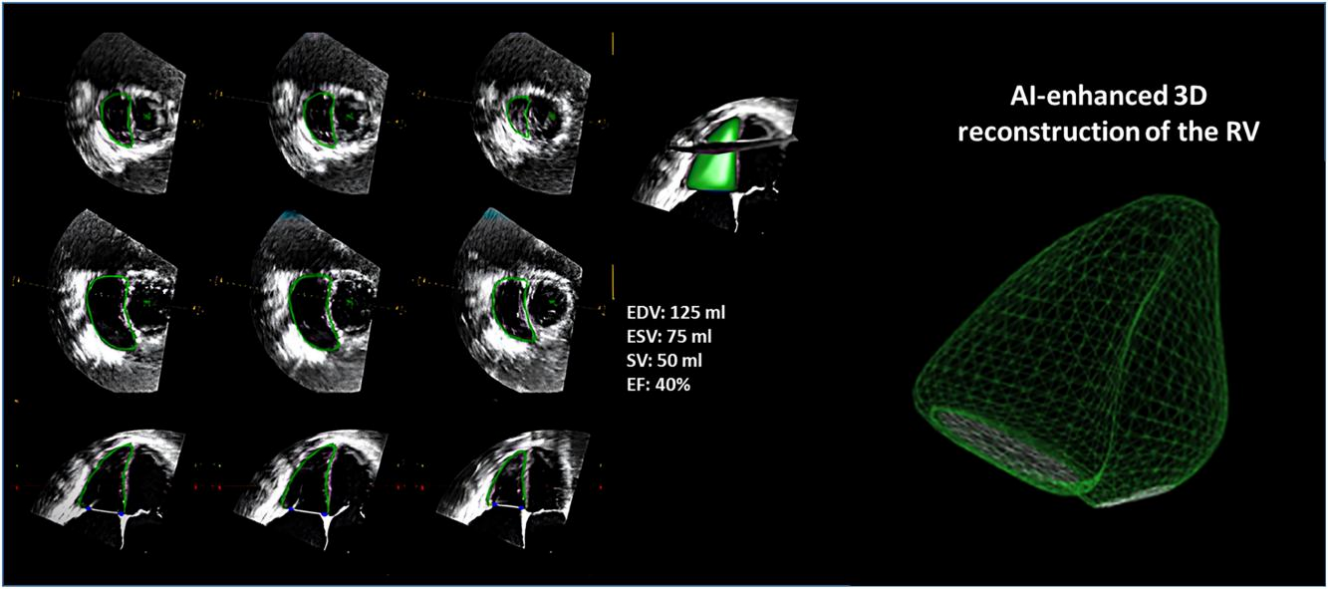
Example of AI-enhanced cardiac chamber quantification with echocardiography. The image displays multi-plane echocardiographic views of the RV with AI-enhanced automatic volumetric analyses.

**Figure 2:**
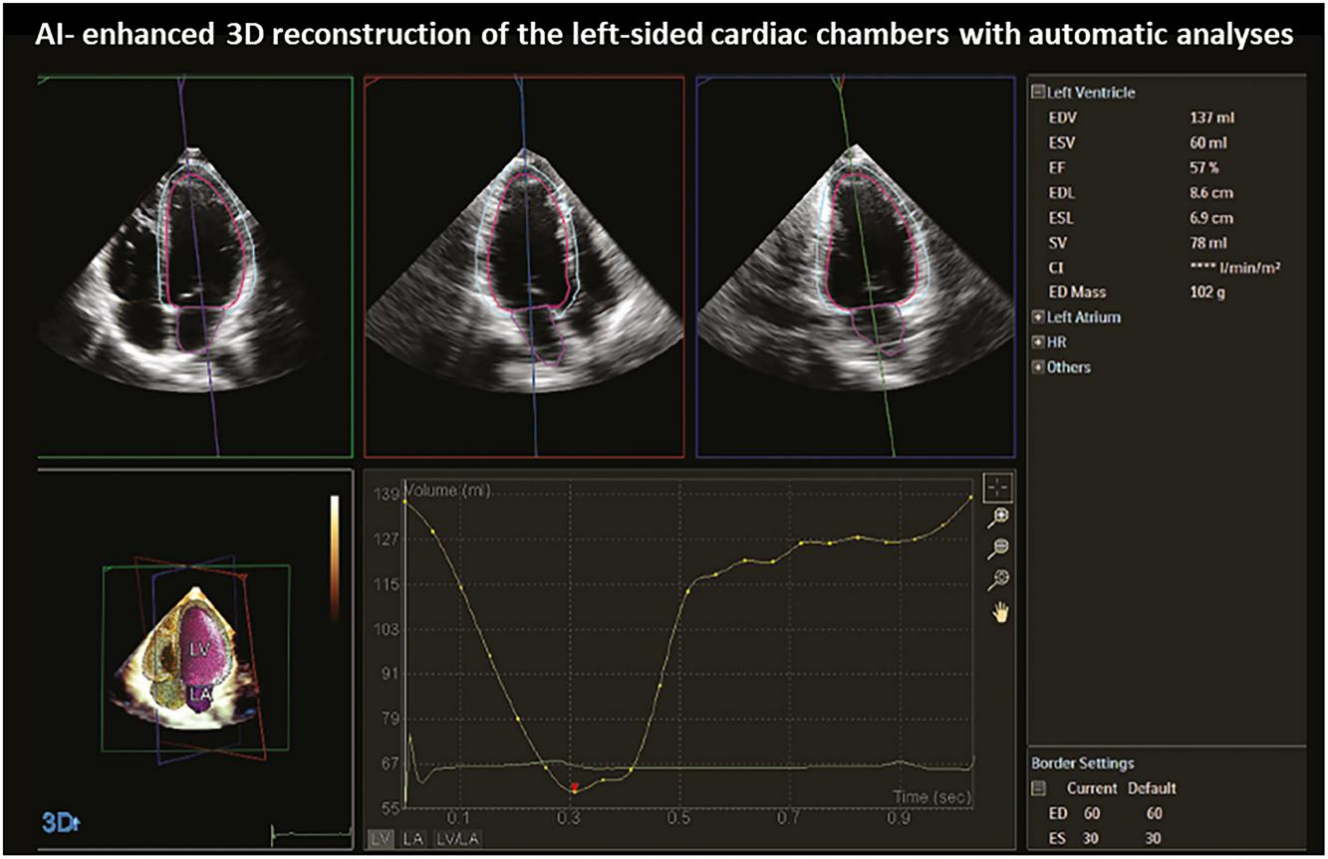
Example of AI-enhanced functional analysis with echocardiography. AI-enhanced automatic 3D reconstruction of the left side of the heart providing quantitative data, including end-diastolic volume, end-systolic volume, EF, and other measurements critical for assessing cardiac function.

AI has also demonstrated a promising role in the quantitative analysis of cardiac volumes and function both for the left and right cardiac chambers (*Figures 1* and *2*). Many studies showed a high degree of accuracy and feasibility in measuring key cardiac parameters such as left ventricular (LV) ejection fraction (EF),^8^ diastolic function,^9^ chamber volumes,^10^ and wall thickness.^11^ With recent advancements, new AI models, following specific training, can even go further, and can recognize complex structural patterns specific for a certain cardiac disease. In 2023, Li *et al*.^12^ managed to successfully establish an automatic deep learning model that correctly differentiated the major aetiologies of increased LV wall thickness, including cardiac amyloidosis, hypertrophic, and hypertensive cardiomyopathy. Many other AI algorithms had been subsequently trained to automatically identify features indicative of other cardiac pathologies such as valvular heart disease,^13^ heart failure,^14^ and constrictive pericarditis^15^ often achieving a level of diagnostic accuracy comparable to that of an expert cardiologist. All these studies highlighting how AI can match or even surpass human accuracy, may lead to a more standardized evaluation, with very limited inter-observer variability. Furthermore, AI’s ability to analyse large datasets quickly allows for the identification of patterns that might be overlooked by human eye. This can be particularly beneficial in identifying sub-clinical conditions or early stages of disease. For instance, Jin *et al*.^16^ found that AI could detect mitral valve prolapse from 3D echocardiographic images with greater sensitivity compared with conventional approaches. Concerning valvular heart disease, AI is transforming the way we assess it by enhancing 3D visualization of heart valves and providing automatic or semi-automatic quantitative measurements of key parameters, such as annular dimensions in mitral regurgitation and quantitative parameters of disease severity. While AI speeds up the grading of valvular heart disease severity, its ability to accurately measure key structures, like the annulus, greatly improves pre-procedural planning, particularly for interventional procedures, ensuring more precise and effective treatments.

AI had also been applied to enhance advanced echocardiographic techniques, such as speckle-tracking echocardiography. Speckle tracking is a technique that tracks the motion of speckles within the myocardium, providing valuable information about myocardial strain and deformation.^17^ This technique provides detailed insights into myocardial mechanics and is crucial for the early detection of even subtle cardiac anomalies. Speckle tracking is a second level assessment, which requires more sophisticated equipment, expert operators and longer time compared with standard exams.^17^ A study conducted by Deng *et al*.,^18^ utilizing a convolutional neural network (CNN) that automated the tracking of myocardial strain not only reduced the time required for analysis but also improved the reproducibility of the measurements. Similar results, achieved in equally complex scenarios such as 3D echocardiography,^19^ colour Doppler, and vector flow mapping,^20^ suggest that AI could make even the most sophisticated echocardiographic techniques more accessible in routine clinical practice.

In the near future, the implementation of machine and deep learning algorithms into ultrasound machines will integrate several new approaches in a seamless manner, potentially removing the need for manual tracing and performing complex scan in less than a minute, without the need of human inputs to correctly classify cardiac views or perform timing of cardiac events. Overall, the potential implications seem limitless, with the capacity to transform not only diagnostic workflows but also risk stratification. A recent study^21^ aimed to estimate age and sex of the patient using standard transthoracic echocardiography, demonstrating both an excellent age estimation with a mean error of 4.9 years and an overall accuracy of 96% for sex prediction. Moreover, the study revealed that an age prediction exceeding 5 years compared with chronological age was associated with an independent 34% increased risk of death during follow-up. While the integration of AI into echocardiography is promising, several challenges remain. One of the primary concerns is the variability in data quality and the generalizability of AI models. Echocardiographic images can vary significantly depending on the machine settings, patient characteristics, and operator experience, so models trained on data from specific dataset may not perform as well when applied to different settings limiting their applicability in clinical practice. Ultimately, AI cannot replace the expertise of sonographers and cardiologists, but it can assist and accelerate their work by enhancing image acquisition and interpretation. Additionally, AI serves as a valuable tool in training the next generation, helping young professionals refine their skills in both acquiring and interpreting echocardiographic images.^1,6^

Key AI contributions in Echocardiography:

- AI enables automated echocardiographic image acquisition, reducing variability, and improving quality.
- Advanced AI models enhance cardiac function analysis and disease detection accuracy.
- AI streamlines specialized techniques like speckle tracking, increasing accessibility, and reproducibility.

## Cardiac magnetic resonance imaging

**Figure 3:**
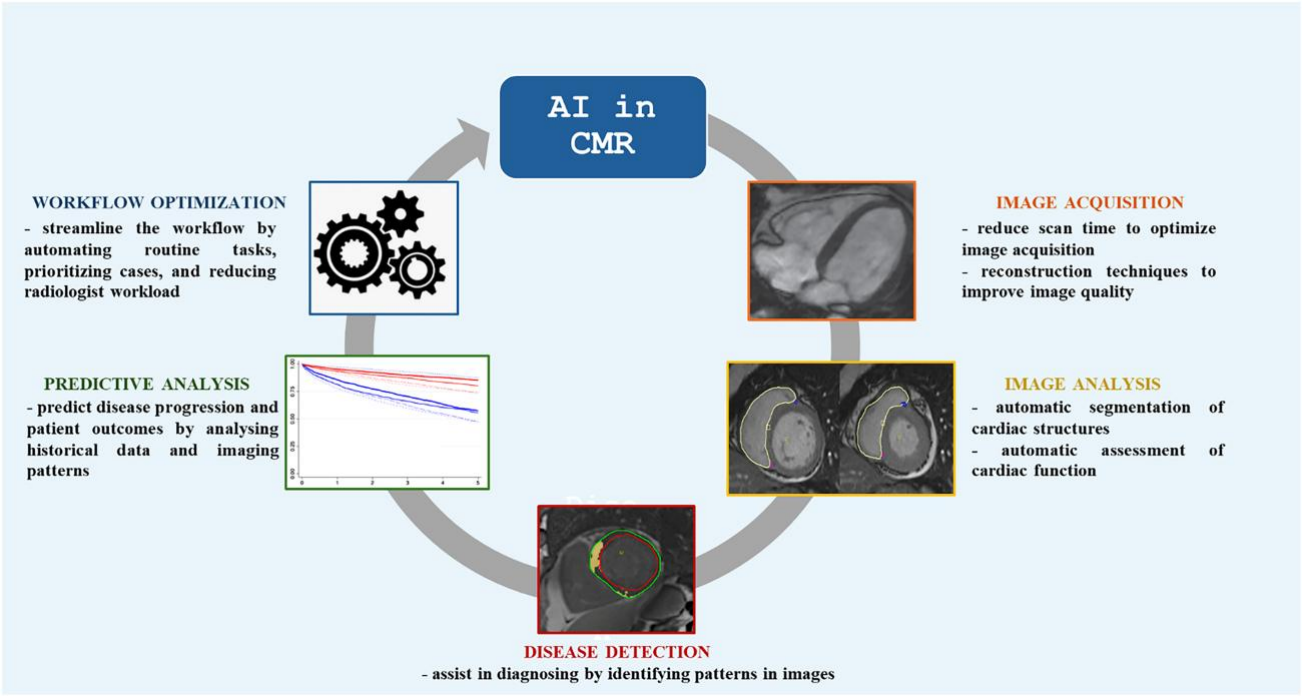
AI applications in CMR imaging. Overview of AI applications in CMR imaging. The figure demonstrates how AI enhances CMR by reducing scan time, accelerating the image reconstruction process, and improving the detection of cardiovascular diseases. Additionally, AI supports risk prediction, offering valuable insights for clinical decision-making and patient management.

CMR imaging plays a pivotal role in the assessment of cardiovascular diseases, providing unparalleled insights into tissue characterization, functional assessment, and anatomical detail without the use of ionizing radiation. Recent years have seen the integration of AI into CMR, driving significant advancements that have enhanced various aspects of image acquisition, processing, analysis, and interpretation (*Figure 3*).

AI has significantly enhanced the efficiency and quality of CMR image acquisition and reconstruction. Traditional CMR imaging, known for its lengthy scan times and the requirement for breath-holding, presents challenges, particularly for certain patient populations. To address these issues, AI algorithms, especially deep learning models, have been developed to accelerate image acquisition and improve reconstruction processes. For example, CNN had been successfully applied to reconstruct high-quality images from under-sampled data, enabling faster scans while maintaining diagnostic accuracy.^22^ Additionally, the combination of compressed sensing techniques with AI-driven reconstruction had facilitated real-time CMR imaging, which is especially beneficial in dynamic studies such as cine imaging.^23^ These advancements not only improve patient comfort but also expand the accessibility of CMR, potentially allowing for broader clinical application.

The automation of cardiac structure segmentation is another area where AI has had a substantial impact. Manual segmentation of CMR images is labour-intensive and prone to inter-observer variability, which can affect the accuracy of subsequent analyses. AI-based segmentation tools, particularly those utilizing deep learning, demonstrated exceptional accuracy and consistency in delineating cardiac chambers, myocardium, and blood pools. Notably, U-net, a widely used deep learning architecture, had been employed to segment the LV and right ventricles (RVs) and myocardium from CMR images with high precision.^24^ These AI models are being trained on extensive datasets, to improve generalizability across different populations and scanner types aiming at effective integration of AI in cardiac imaging and clinical cardiology.^25^ The automation of segmentation through AI not only saves time but also aims at enhancing the precision of volumetric measurements, strain analysis, and tissue characterization, which are critical for diagnosing and monitoring cardiovascular diseases.

AI’s role in myocardial tissue characterization is particularly noteworthy, especially in the detection and quantification of fibrosis, oedema, and infarction. Traditional techniques such as late gadolinium enhancement (LGE) and T1/T2 mapping have been significantly enhanced by AI-driven approaches, which improve the accuracy and efficiency of tissue characterization. AI algorithms have been developed to automatically detect and quantify areas of fibrosis and scar tissue from LGE images, providing more objective and reproducible assessments than manual interpretation both in ischaemic and non-ischaemic cardiomyopathies.^26,27^ Furthermore, machine learning (ML) models are capable of differentiating between various myocardial pathologies by analysing texture and intensity features extracted from CMR images.^4^ These advancements allow for more precise phenotyping of cardiomyopathies and ischaemic heart disease, which is crucial for tailoring patient management and predicting outcomes.

AI has also demonstrated significant potential in detection and prognostication of cardiovascular diseases using CMR. ML models trained on large datasets can identify patterns and features in CMR images that may indicate disease.^28^ Furthermore, AI-driven risk stratification tools can integrate CMR data with clinical parameters to provide personalized prognostic insights for patients with coronary artery disease (CAD) and other cardiovascular conditions.^29^ These predictive models are invaluable in guiding clinical decision-making, enabling earlier interventions and more targeted therapies that can improve patient outcomes.

Since the role of CMR in cardiovascular health is growing, much effort has also been put in creating deep learning frameworks aiming to automating the entire scanning procedure, from protocols prescription to imaging parameters selection in order to compress the scanning time and allow CMR technologists to supervise and focus on quality control more.^30^

Looking forward, the integration of AI in CMR is likely to involve greater fusion with multimodal imaging and big data analytics. Combining CMR with other imaging modalities, such as positron emission tomography (PET) or CT, can provide a more comprehensive assessment of cardiovascular health. AI can facilitate the synthesis of data from these diverse sources, enabling more accurate and holistic evaluations of complex cardiovascular conditions. Despite these advancements, several challenges remain in the widespread adoption of AI in CMR. These include the need for large, diverse datasets to train robust models, ensuring the generalizability of AI tools across different populations and imaging centres, and addressing ethical and regulatory considerations related to AI in healthcare.

Key AI contributions in CMR:

- AI accelerates CMR imaging, improving acquisition speed and diagnostic accuracy.
- Automated segmentation enhances precision in cardiac structure and tissue characterization.
- AI-driven risk models integrate CMR data for personalized disease detection and risk stratification.

## Cardiac computed tomography

**Figure 4:**
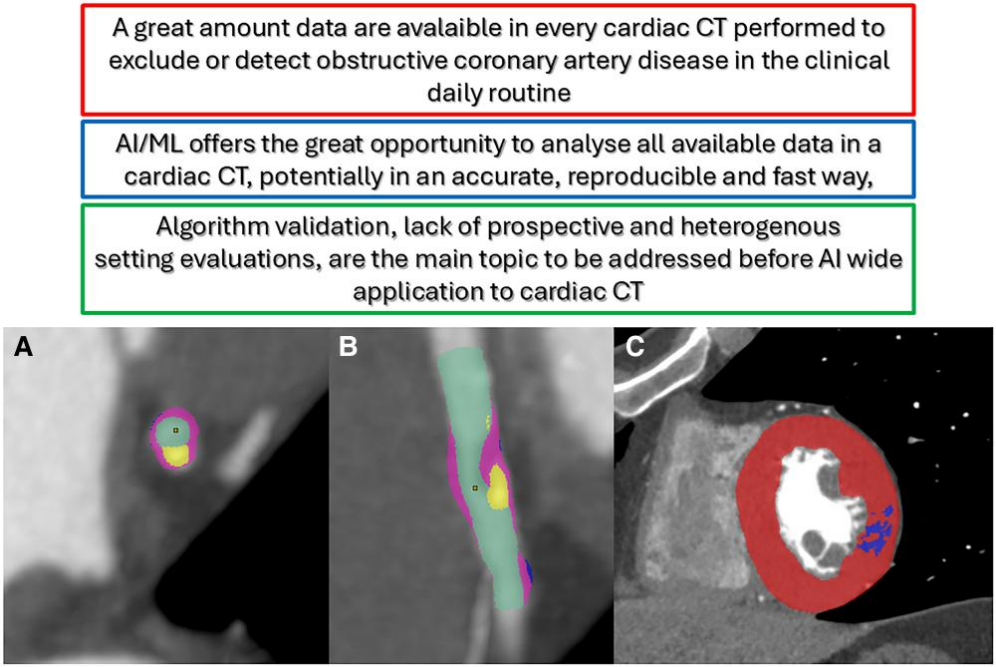
Challenges and opportunities related to AI implementation in CCT. The figure presents the ‘status quo’ (red box), ‘opportunities’ (blue box), and ‘unsolved challenges’ (green box) related to the use of AI in CCT. In Panel *A*, a short-axis view illustrates a mixed plaque with calcified (yellow) and fibro-lipid (purple) components. Panel *B* shows a long-axis view of the same plaque. Panel *C* highlights a LV short-axis view of a myocardial perfusion defect (blue). Each panel includes examples of quantitative CT data analysis, showcasing areas where AI could significantly enhance the evaluation and interpretation of imaging results. ML, machine learning.

Several potential applications of AI in CCT have been recently described. The entire workflow of CCT, from patient selection to advanced image analysis and clinical decision-making could be implemented by AI (*Figure 4*). Previous studies focusing on CCT image acquisition and reconstruction have demonstrated that AI can reduce radiation exposure by optimizing acquisition protocols and improving reconstruction algorithms, thereby enhancing overall image quality.^31,32^

The main applications of AI for CCT are related to the detection and characterization of CAD. Recently, the CLARIFY (CT EvaLuation by Artificial Intelligence For Atherosclerosis, Stenosis and Vascular MorphologY) study showed that AI-based coronary CT angiography (CCTA) assessment had high diagnostic performance in detecting significant stenosis (>50%) with a sensitivity and specificity of 80 and 97%, respectively, when compared with results from expert readers.^33^ Of interest, CCT had been recently recognized as the sole non-invasive tool for the evaluation of coronary atherosclerosis that can represent a surrogate end-point of interest for clinical studies. As a consequence, considering that a complete analysis with the quantification of coronary total plaque volume using a conventional, non-AI-enhanced approach may take up to 30 min,^34^ it is crucial to improve reproducibility and reduce the time required for these advanced analyses. On this regard, the prospective, multi-centre study to analyse PLAQUE study recently demonstrated that AI-enabled CCTA quantification of coronary atherosclerosis is strongly associated with intra-vascular ultrasound (IVUS) data, which is the gold standard for the assessment of coronary atherosclerosis.^35^ This automation accelerates the interpretation process, allowing clinicians to focus on more complex tasks. However, it should be acknowledged that not all studies on AI applied to CCT evaluation had positive results. In a sub-study of the CLARIFY trial, an AI-based software was tested in 232 patients for detecting high-risk plaque features, including positive remodelling and low-attenuation plaque. This study revealed poor agreement between the AI-based system and 3 expert readers, with weighted Kappa coefficients of 0.22, 0.17, and 0.26, respectively.^36^

Beyond CAD, AI, and ML can automatically segment cardiac structures like the ventricles, atria, and vessels in both contrast-enhanced and non-contrast CT images. These algorithms accurately define the boundaries of these structures, enabling precise measurements of ventricular volumes, EF, and myocardial mass. This certainly finds practical application in the pre-procedural CCT assessment for structural interventional procedures, such as transcatheter aortic valve implantation, where AI-based algorithms showed to be extremely useful for pre-procedural planning.^37^

Despite the promising advancements, several challenges persist in implementing AI in CCT imaging. Similar to the implementation of AI for the other cardiovascular imaging modalities, a key obstacle is the need for large, diverse, and well-annotated datasets to train AI algorithms effectively. The quality and generalizability of AI models hinge on access to robust datasets that cover diverse patient populations and imaging scenarios. Additionally, regulatory approval and thorough clinical validation are crucial to guarantee the safety and efficacy of AI tools in real-world clinical practice. Concerns also exist around the interpretability of AI models in CCT, as some function as ‘black boxes’, making it difficult for physicians to understand the reasoning behind diagnoses or recommendations. Ensuring transparency and fostering trust in AI-based diagnostic tools applied to CCT data are essential for their broader acceptance and use in diverse clinical settings. Finally, one of the most fascinating future challenges of the AI use in CCTA would be the prediction of plaque instability and the occurrence of acute coronary syndromes; however, data on this task are still limited.

Key AI contributions in CCT:

- AI enhances CCT image quality, reduces radiation, and optimizes acquisition protocols.
- AI improves CAD detection and quantifies atherosclerosis accurately.
- AI automates segmentation of cardiac structures for precise pre-procedural planning of structural procedures.

## Interventional cardiology imaging

**Figure 5:**
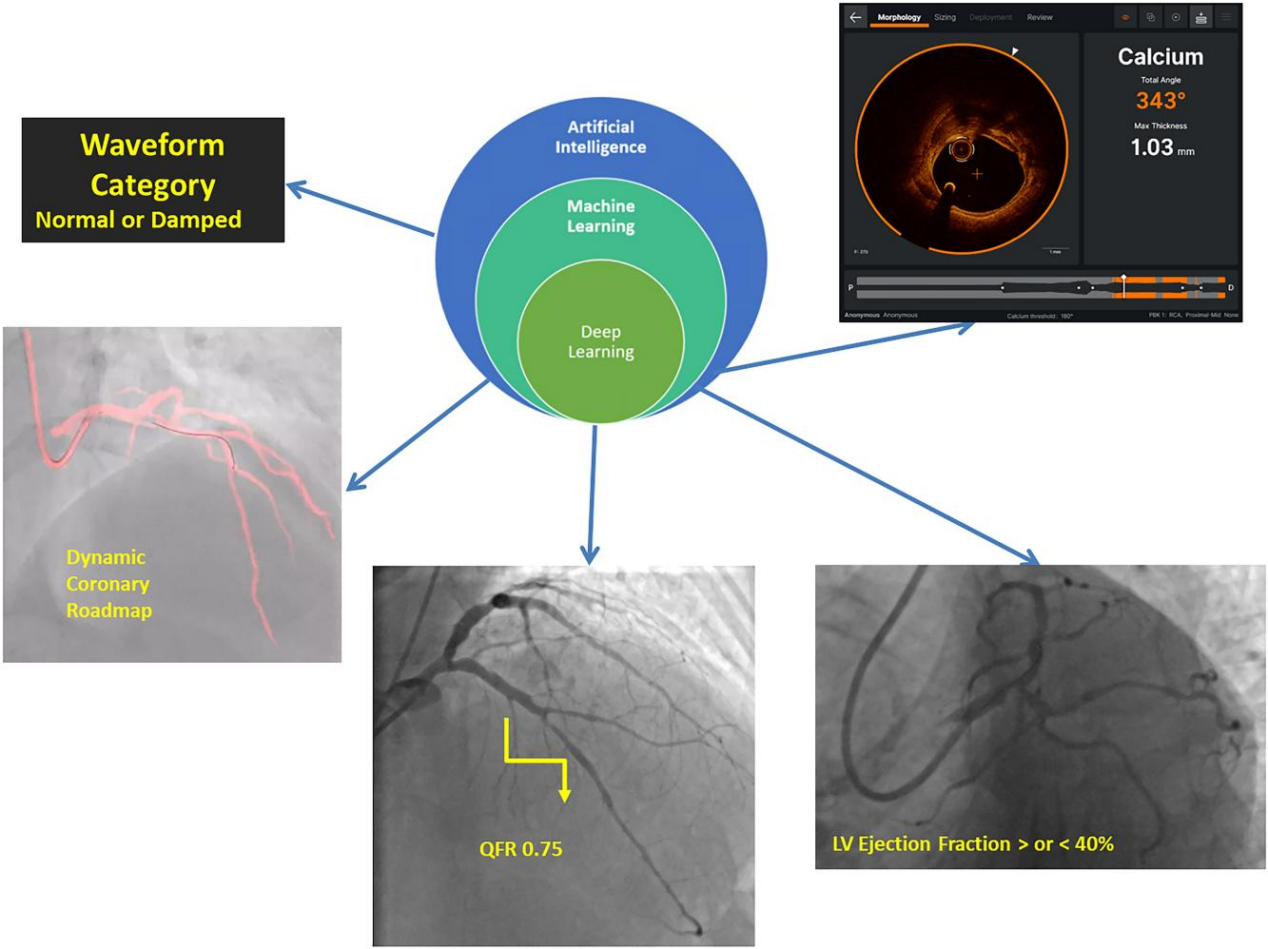
Overview of AI applications in interventional cardiology imaging. The figure highlights AI's role in detecting pressure wave damping during CAG, automating plaque characterization, defining coronary anatomy, and identifying LV systolic dysfunction from standard CAG images. These advancements demonstrate AI’s potential to enhance accuracy and efficiency in interventional cardiology imaging. QFR, quantitative flow ratio.

AI, especially its sub-field of deep learning, is revolutionizing interventional cardiology with several applications (*Figure 5*). In this field, AI-enhanced assessment can increase diagnostic accuracy and risk stratification especially in CAD. Coronary angiography (CAG) is the gold standard for CAD diagnosis and provides the data on which percutaneous coronary interventions (PCI) are planned and performed. However, CAG interpretation is notably operator dependent and deep learning showed to be useful to develop coronary artery diagnostic map^38^ that can limit inter- and intra-observer variability and enhance interventionalist interpretation. This deep learning algorithm called ‘Deep Discern’ was trained to correctly recognize and label coronary artery segments and morphologies of lesions (i.e. stenosis, calcification, dissection, and thrombosis).^38^ ML technology had been also used to accurate recognize damping of aortic pressure during CAG representing a promising tool to assist operators and guarantee patient safety.^39^ Concerning the functional assessment of coronary artery stenosis during CAG, fractional flow reserve (FFR) is the gold standard. Unfortunately, FFR is not widely used in clinical practice because it requires the use of adenosine to induce hyperaemia and the insertion of a pressure wire into the coronary artery of interest potentially increasing the duration of the procedure and making it somehow more complex. Quantitative flow ratio (QFR) is a computationally calculated estimation of FFR based on automated AI assessment of conventional CAG images that does not require the use of pressure wire and adenosine.^40^ QFR demonstrated to be a feasible alternative to FFR and has been introduced for the first time into the last European guidelines on chronic coronary syndromes.^41^ QFR is based on 3D quantitative CAG analysis and modified frame counting for estimating coronary flow velocity and requires just two conventional CAG projections of at least 20° apart.^40^

Concerning the use of AI in intra-vascular imaging, the current clinical use and interpretation of IVUS, which is the most used intra-coronary imaging technique, is limited by cardiac motion artefacts especially in the longitudinal view. Recently, Erdogan *et al*.^42^ proposed to analyse end-diastolic IVUS frames using deep-learning methodology overcoming its current limitations and obtaining higher reproducibility on the assessment of CAD. Intra-vascular coronary imaging is also recommended after PCI and need to be considered in case of angiographic ambiguous stenosis. AI had been widely implemented in currently used optical coherence tomography (OCT) software. The use of this software allows automatic measurement of the lumen area and diameters that are of pivotal importance during PCI to correctly choose stent and balloon dimensions. Moreover, Chu *et al*.^43^ developed an AI-based tool to characterize atherosclerotic plaques automatically from OCT data. This tool is based on a CNN and showed excellent detection of fibrous, lipid, and calcific plaques with a diagnostic accuracy of 98, 91, and 89%, respectively.^43^ Regarding the use of intra-vascular imaging after PCI, this is limited by the time and expertise needed to analyse the struts to detect complications such as malposition, under-expansion, plaque protrusion, or edge dissection. AI showed to be extremely useful for this task. Wu *et al*.^44^ developed a CNN tool with pseudo-3D input and shortcut connections to join information from adjacent OCT frames. This model demonstrated excellent performance for the detection and segmentation with high analysis speed compared with previous semi-automatic methods^44^ and allowed 3D stent rendering facilitating the detection of complications after PCI. AI-enhanced software can also provide real-time, automatic, motion-compensated coronary imaging for live guidance of wires and intra-coronary devices^45^ reducing contrast volume, procedural time and the use of ionizing radiation.

CAG with the help of AI can provide data, which are nowadays neglected. For instance, Rostami *et al*.^46^ used a combined 3D-CNN and transformer to correctly assess LVEF from CAG data. This calculation is based on the displacement of the epicardial coronary arteries in various views and outperformed human expert to detect patients with LV systolic dysfunction (LVEF < 40%).^46^ One of the most fascinating future application of AI in interventional cardiology would be its use to integrate CAG and imaging data with clinical data and biomarkers to predict the best option between PCI and optimal medical therapy in chronic coronary syndromes.

Key AI contributions in Interventional Cardiology:

- AI enhances diagnostic accuracy, reduces variability, and aids coronary artery assessment by CAG.
- AI enables FFR alternatives like QFR and improves intra-vascular imaging analysis.
- AI provides real-time guidance, detects complications, and predicts treatment outcomes in patients undergoing coronary intervention or structural heart procedures.

## Other applications in cardiovascular imaging

In addition to its applications in echocardiography, CMR, CCT, and CAG, AI also plays a significant role in other areas of cardiovascular imaging such as nuclear cardiology and EP, further expanding its impact on cardiovascular care.

### AI in nuclear cardiology

Recently improvements in PET, and single-photon emission computed tomography (SPECT), have resulted in increased diagnostic efficiency. Nonetheless, human interpretation of images and quantification of these datasets are limited. AI in nuclear cardiology might be implemented along all the steps of the process, from image acquisition and reconstruction to image analysis, disease reclassification, and risk stratification. In this scenario AI had been mostly studied for the evaluation of critical CAD probability and had also been integrated with clinical data to further improve risk stratification. A recent study of 1181 patients found that ML provided with SPECT myocardial perfusion imaging and clinical data showed higher area under the curve (AUC) (0.94 ± 0.01) than total perfusion deficit (0.88 ± 0.01) or visual read out, for the detection of critical CAD as defined by CAG.^47^ A large analysis of 2619 patients evaluating clinical characteristics, stress test, and SPECT imaging variables demonstrated a high predictive accuracy of the ML algorithm for 3-year risk of major adverse cardiovascular events (AUC = 0.81 [0.78, 0.83]), which was superior to existing visual or automated perfusion assessments.^48^ However, current evidence on ML capabilities has primarily been validated through retrospective studies, particularly by integrating clinical data with nuclear imaging data to detect obstructive CAD. It remains unclear whether ML models can outperform existing predictive models or scores in prospective settings. Therefore, prospective validation in adequately powered randomized controlled trials is essential to determine its true impact on clinical practice.

### AI in EP imaging

**Figure 6:**
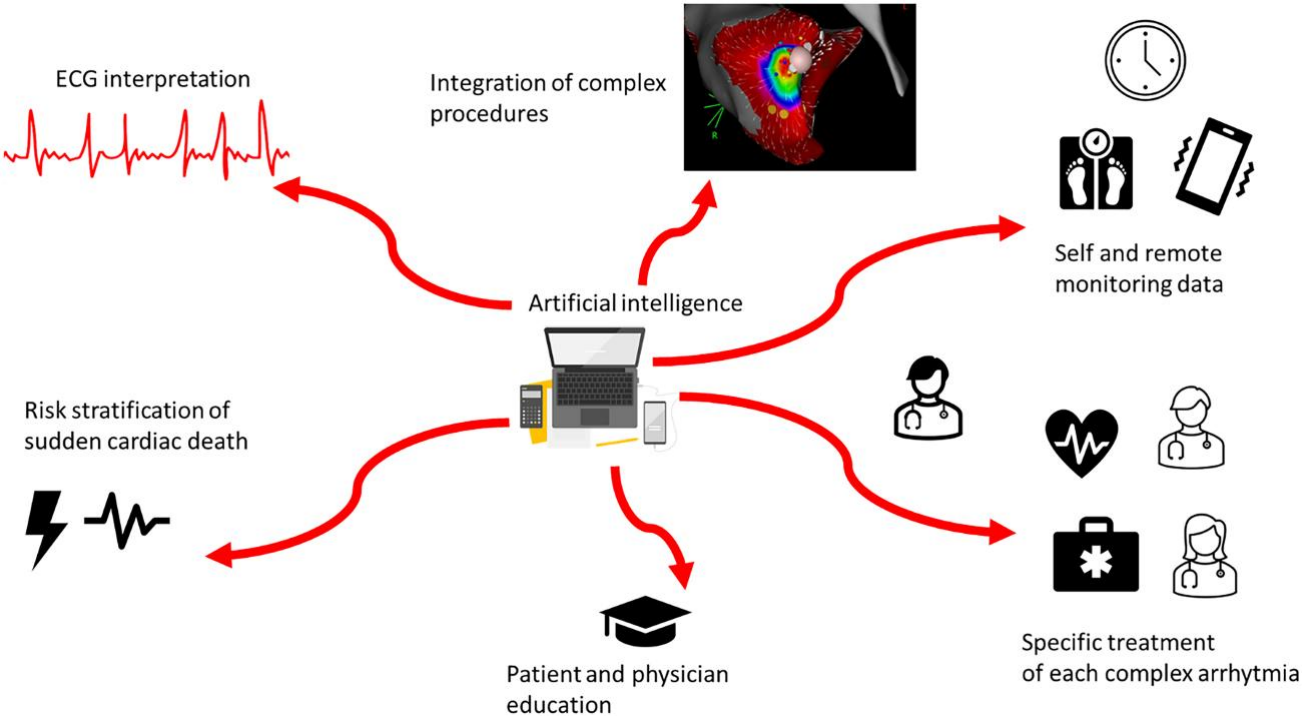
AI applications in the imaging field of EP. The figure highlights AI’s role in data integration, risk stratification, procedural guidance, automatic ECG interpretation, treatment optimization, and education in the field of EP.

EP is one of the most rapidly evolving branches of medicine. Every day, electrophysiologists encounter new challenges, particularly due to the increasing use of telemedicine, which generates huge amounts of data. AI has numerous potential applications in EP, including improving electrocardiogram (ECG) interpretation, aiding in risk stratification for sudden cardiac death, integrating data from remote monitoring, enhancing the execution of EP procedures, personalizing patient treatment based on individual profiles, and supporting the training of both physicians and patients (*Figure 6*). Furthermore, AI could serve as a valuable tool for patients, helping them better understand their condition, its progression, and the importance of adhering to therapies and follow-up care. This, in turn, can foster greater engagement in managing their disease. AI has the potential to become a cornerstone in the future of arrhythmia care delivery. The ECG is one of the oldest diagnostic tests available and is routinely used worldwide. AI could play a significant role in enhancing ECG interpretation, as well as in risk stratification, disease screening, and the detection of uncommon conditions. Remote monitoring, along with smart and wearable devices, allows vast amounts of data to be routinely available for both patients and physicians. AI algorithms applied to ECG data demonstrate high accuracy in identifying cardiac arrhythmias including atrial fibrillation. Imaging is a fundamental part of any EP procedure. The routine uses of 3D electro-anatomic mapping systems (EAMs) has drastically changed daily clinical practice in EP labs by significantly reducing the use of fluoroscopy. Some studies explored the use of extended reality technologies, such as virtual reality, which creates a fully virtual environment, and augmented reality, where real-time images of the procedure are overlaid with previously acquired scans (e.g. CMR or CCT) enhancing interpretation and clinical decisions. These techniques could be revolutionary, particularly in improving visualization of non-standard human anatomy. A small, preliminary study involving 10 patients undergoing catheter ablation^49^ explored the use of an AI-based augmented reality system that integrated EAM of the arrhythmia with pre-procedural CT scans. This system also provided real-time visualization of catheter movement, enhancing procedural guidance and facilitating the ablation process. AI is expected to play a major role in EP in the coming years by enhancing our understanding of disease, improving risk stratification, and facilitating the interpretation of ever-increasing amounts of data. Additionally, the integration of AI to combine and analyse imaging data from multiple modalities is anticipated to further advance EP procedures, improving both accuracy and outcomes in patient care.

Key AI contributions in Nuclear Medicine and EP:

- AI improves image analysis, risk stratification, and CAD detection in nuclear cardiology.
- AI enhances ECG interpretation, arrhythmia detection, and remote monitoring in EP.
- AI can help the integration of imaging and EP data to guide interventional EP procedures predict and improve their outcomes.

## Conclusions

AI is poised to revolutionize cardiovascular imaging, offering significant benefits in terms of efficiency, accuracy, and integration of complex data across various imaging modalities. Given its wide range of applications cardiovascular imagers should embrace AI as a powerful tool and integrate it into their clinical practice. However, it is essential to recognize that AI will not replace the role of physicians. Instead, it will enhance workflow efficiency, allowing clinicians to focus more time and expertise on complex cases. Understanding AI potential, while acknowledging its limitations, will be key to harnessing its full value in advancing patient care.

## Data Availability

No new data were generated or analysed in support of this research.

## Lead author biography

Dr. Fortuni graduated with honours in Medicine and Surgery from the University of Perugia and specialized in Cardiology at the University of Pavia. He completed an advanced cardiovascular imaging fellowship at the University of Leiden, Netherlands. Dr. Fortuni has been recognized for his contributions to clinical cardiology, winning the Young Investigator Award in Clinical Cardiology from the European Society of Cardiology in 2020 for a project on secondary tricuspid regurgitation. He also won the Young Investigator Award of the Italian Society of Cardiovascular Imaging. With over 100 peer-reviewed publications in high-impact international journals, Dr. Fortuni focuses on multimodality imaging in valvular heart disease and heart failure. As a reviewer for leading cardiology journals, he was named an Outstanding Reviewer by *Circulation* in 2020 and *JASE* in 2023. Dr. Fortuni serves on the editorial boards of *JASE*, *Frontiers in Cardiovascular Medicine*, and *Cardiovascular Ultrasound*, and is the Associate Editor for *European Heart Journal: Imaging Methods and Practice*. Currently, Dr. Fortuni is a consultant cardiologist specialized in cardiovascular imaging at the Heart Failure Unit of S. Maria della Misericordia Hospital (Perugia, Italy) and a faculty member at the University of Perugia (Italy).
